# Supercritical fluid extraction of β-carotene from ripe bitter melon pericarp

**DOI:** 10.1038/s41598-019-55481-4

**Published:** 2019-12-17

**Authors:** Avinash Singh Patel, Abhijit Kar, Sukanta Dash, Sanjaya K. Dash

**Affiliations:** 1 Division of Food Science and Postharvest Technology ICAR – Indian Agricultural Research Institute New Delhi, New Delhi, 110012 India; 2 Division of Design and Experiments, ICAR – Indian Agricultural Statistics Research Institute, New Delhi, 110012 India; 3 Division of Agricultural Processing & Food Engineering, College of Agricultural Engineering & Technology, Orissa University of Agricultural & Technology, Bhubaneswar, 751003 India

**Keywords:** Chemical modification, Drug delivery, Drug development

## Abstract

Study ascertained the recovery of β-carotene from enzyme-treated (enzyme load of 167 U/g) pericarp of ripe bitter melon using supercritical fluid extraction (SFE) technique. Effect of different pressure (ranged from 150–450 bar), carbon dioxide (CO_2_) flow rates (ranged from 15 to 55 ml/min), temperatures (from 50 to 90 °C), and extraction periods (from 45–225 minutes) were observed on the extraction efficiency of β-carotene. Results showed that extraction pressure (X_1_) among extraction parameters had the most significant (p < 0.05) effect on extraction efficiency of the β-carotene followed by allowed extraction time (X_4_), CO_2_ flow rate (X_2_) and the temperature of the extraction (X_3_). The maximum yield of 90.12% of β-carotene from lyophilized enzymatic pretreated ripe bitter melon pericarp was achieved at the pressure of approx. 390 bar, flow rate of 35 mL/min, temperature at 70 °C and extraction time of 190 min, respectively. Based on the accelerated storage study the 70% retention shelf life of the β-carotene into extract was estimated up to 2.27 months at 10 °C and up to 3.21 months at 5 °C.

## Introduction

Increasing the demand for natural colorants, concerning the derivation of researchers has rewarded the attention towards new biological resources instead of attention to chemical synthesis^1–6^. Several natural colors such anthocyanin, carotenoids, chlorophyll, betalains, iridoids, phycobiliproteins, etc. are extensively have been studied for their potential as a natural food colorant^2,6–9^. Moreover, these natural colorants have nutraceutical properties, which helps to fight against several diseases such as cancer, cardiac, inflammation, diabetes, neural problems, etc.^1,6^. Carotenoids are the yellowish-red pigments found in many plants, algae, and phototropic bacteria and have attracted vast research attention in the global market due to their potent antioxidant properties^8,10,11^. Beta-carotene (β-carotene) is one of the extensively used carotenoids as either additives or dietary supplements since years and helps in preventing several types of cancer including, lung, stomach and skin^12–15^. It is rehabilitated in the human body as a precursor of vitamin A (retinol) that is indispensable for appropriate function of the retina, epidermis and mucous membranes^16^ and providing other health benefits, including the possible prevention and treatment of cardiovascular disease^15,17^.

At present the commercial production of the β-carotene is done by either chemical synthesis using β-ionone or from limited selective natural resources^2,6,10,18^. Among the natural sources of the beta-carotene viz., *Dunaliella* a green microalgae contains 2–3 g of beta-carotene per litre^19^, carrot contains 110 µg per 100 g of fresh weight^20^ and *Flavobacterium multivorum* a bacteria contains 7.85 µg per milliliter^10^ are the most commercial and widely used.

Bitter melon (Momordica charantia L.) is a climbing plant of *Momordica* genus mostly grown in Asian, African and Caribbean countries that have been used for various curative purposes^21^. The outer layer of the fruit is rough, known as pericarp, inner smooth tissue is an appendage or covering of seed called as aril. After ripening bitter melon, fruits turn to yellow due to their rich in carotenoids. Cultivation of ripe fruits at an industrial scale as well as farmer level is only for seed production however the waste and by-products generated during seed processing constitute a great source of β-carotene (967 µg/100 g fresh weight), can be a potential for the commercialization for the beta-carotene production^22,23^.

Traditionally the extraction process of valuable compounds from agricultural produces is usually performed by using organic solvents; however, this chemical method may be toxic and has some pollution concerns^24^. One alternative to traditional extraction by organic solvents is to accomplish the extraction by CO_2_ based supercritical fluid extraction (SFE) method, which is used to extract numerous bioactive compounds due to its inflammable, protective solvent in nature that responsible for an anaerobic extraction results better stability than the organic solvent extraction^25,26^. The application of SFE technique to recover the essential compounds is more effective than other techniques, especially considering environmental protection. Moreover, eliminate the process of after separation from extracts resulting extensively pure^27^. β-Carotene extraction with SFE from tray dried carrots is an excellent technique suitable to replace the use of harmful organic solvents and satisfy the increasing demand for biological solvent-free β-carotene^20^. The extraction efficiency of targeted compounds depends upon the applied extraction parameters such as extraction pressure and temperature that both are significant SFE equipped constraint. In both indispensable characteristics of supercritical fluid, other factors such as the interaction between targeted compounds and ecological aspects are also important to extraction efficiency^28^. Due to these multi-faceted relations, a solitary condition of SFE cannot give sufficient information. To defeat this complexity, response surface methodology (RSM) a statistical investigational design has frequently been implemented to assist seems to be the optimum process factors^29,30^. Except for instrumental parameter, other factors such as enzymatic digestion, heat treatment, particle size, moisture content matrices play a key role in the extraction efficiency of targeted compounds^31,32^. These pretreatments may humiliate the complex structure of matrixes with targeted compounds and make it easily available and improve the content also by conversion process^33,34^. Since the call wall of plants is composed of main polysaccharides (cellulose, hemicelluloses) and heteropolysaccharide (pectin), most of the bioactive compounds are contained in these saccharides and bind to gather tightly. Enzymes mainly cellulase, pectinase, protease, and α-amylase are used to make extraction efficiency of these compounds^11,34^.

Enzymatically pretreated and solvent added SFE of bioactive compounds were performed by many researchers^34,35^. SFE of β-carotene from different bio-resources were also studied^20,36^. However, very little or no reports are available on SFE of β-carotene from the ripe bitter melon pericarp. Hence, an attempt was made to investigate systematically the effects of SFE parameters on the percent yields of β-carotene from pretreated followed by lyophilized pericarp of ripe bitter melon.

In particular, interest lies in determining the effect of different SFE parameters on the extraction efficiency of beta-carotene from ripe bitter melon pericarp. A response surface methodology (RSM) tool is applied to optimize best set of combination of pressure, temperature, time and flow rate. Extracted beta-carotene is quantified using supercritical fluid chromatography (SFC) technique.

## Materials and Methods

### Materials

Ripe bitter melon was procured from the orchard of Indian Agricultural Research Institute, New Delhi. Chemicals and reagents in this experiment were purchased from Merck, KGaA-64271, Darmstadt, Germany. β-Carotene standard was purchased from Sigma-Aldrich (Chemie GmbH, Taufkirchen, Germany). Pectinase (456 U/g, Aspergillus sp.) was purchased from Sigma-Aldrich (Japan) and deep tube liquid CO_2_ (99.98%) from Amit labs, New Delhi, India.

### Sample preparation

Ripe bitter melon was washed in tap water to remove dust and foreign materials. The top and bottom portion of washed fruit were removed, then the whole fruit was cut one-sided lengthwise with help of knife. The aril with the seed of fruits was removed manually and collected pericarp was kept at −20 °C temperature.

### Enzymatic digestion of pericarp

For the enzymatic pretreatment of the ripe bitter melon pericarp a method described by Ranveer *et al*.^35^ was used. A working enzyme solution of 167 U/g was prepared by diluting the stock enzyme solution into citrate buffer (pH 5.0)^35^. Reaction mixture was prepared in the ratio of 3:1 (enzymatic citrate buffer solution: pericarp). The reaction mixture was continuously stirred at 25 °C for 4 hours. This mixture was filtered through Whatman No. 42, and the residue was kept in a deep freezer (−80 °C e) for 6 hours. The pericarp was lyophilized by Labconco lyophilizer (Kansas, USA) at the temperature of −55 °C with a vacuum of 0.1 mbar for 72 hours. The lyophilizing jars were wrapped with aluminum foil (11 µm thickness) to avoid degradation.

### Supercritical fluid extraction (SFE) of β-carotene

**Figure 1:**
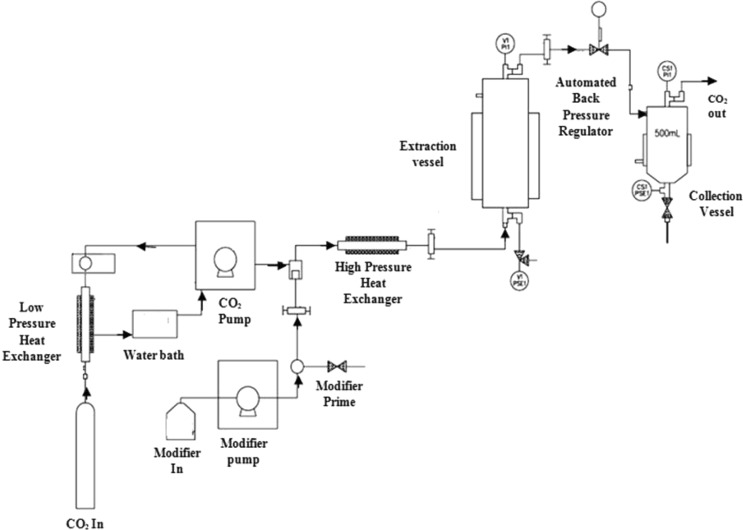
SFE process flowchart used in the extraction of β-carotene from the ripe bitter melon pericarp.

Before feeding the sample in 1 litre steel vessel of SFE, the lyophilized pericarp was powdered and sieved with standard 35 BSS mesh (500 µm pore size). Extraction of β-carotene from powdered pericarp matrices was carried out at each of the experimental combinations through an automated SFE system (Model 7100, Thar Technologies Inc., USA). For each extraction run, the extraction vessel was loaded with 50 g of powdered pericarp matrix. Ethanol (5%, w/w) was used as a co-solvent to enhance the extraction yield^30^. SFE parameters during the study were controlled by software (SuperChrom SFC Suite v5.9, Thar Technologies Inc., USA). The systematic representation of the SFE system for extraction of β-carotene from powdered matrix shown in Fig. 1, which comprises two high-speed pumps, one is CO_2_ pump (280 mL/min) and another is modifier pump (150 mL/min). The extracted β-carotene at the end of each experiment was collected, vacuum concentrated and stored at −20 °C until quantitative analysis.

### Analysis of total β-carotene (TBC)

The extracted β-carotene was characterized quantitatively using supercritical fluid chromatography based ultra-performance conversance chromatography (Acquity UPC^2^ system, Waters Technologies, USA) equipped with a reverse-phase analytical polymeric High Strength Silica C18 (HSS C18 SB), 3 × 100 mm with particle size 1.8 µm, as reported by Runco *et al*.^37^. Empower^3^ software was used to operate the system during the quantitative analysis of samples.

### Experimental layout

For optimization of the extraction parameters, the independent variables were coded as X_1_ (pressure), X_2_ (flow rate), X_3_ (temperature), and X_4_ (time) for maximum recovery of β-carotene using supercritical CO_2_ based SFE technique from ripe bitter melon pericarp matrices (Table 1). The whole experiment was designed to use the central composite design (CCD) of response surface methodology (RSM) resulted in thirty experiments and each was conducted for the optimization studies (Table 2). Data pertaining to five independent and one response variable were analysed to get a multiple regression equation:

1 $$\begin{array}{ccc}Y & = & {b}_{0}+{b}_{i}{X}_{i}+{b}_{j}{X}_{j}+{b}_{k}{X}_{k}+{b}_{l}{X}_{l}+{b}_{ij}{X}_{ij}+{b}_{ik}{X}_{ik}+{b}_{il}{X}_{il}+{b}_{jk}{X}_{jk}+{b}_{jl}{X}_{jl}\\  &  & +\,{b}_{kl}{X}_{kl}+{b}_{ii}{X}_{i}^{2}+{b}_{jj}{X}_{j}^{2}+{b}_{kk}{X}_{k}^{2}+{b}_{ll}{X}_{l}^{2}\end{array}$$

where Y refers to the measured predicted % yield, b_0_ is the intercept; b_i_, b_j_, and b_k_ and b_l_ are the linear terms; b_ij_, b_ik_, b_il_, b_jk_, b_jl_ and b_kl_ are interaction coefficients and b_ii_, b_jj_, b_kk_ and b_ll_ are quadratic terms, respectively.

**Table 1:** Central composite rotatable design (CCRD) showing independent variables, their levels and responses.

Independent variables	Symbols	Coded variable levels				
		−2	−1	0	1	2
Pressure (bar)	X_1_	150	225	300	375	450
Flow rate (mL/min)	X_2_	15	25	35	45	55
Temperature (°C)	X_3_	50	60	70	80	90
Time (min)	X_4_	45	90	135	180	225

**Table 2:** Central composite arrangement for independent variables.

Run	Independent variables levels (coded)				Yield (%)	
	X_1_	X_2_	X_3_	X_4_	Experimental	Predicted
1	−1	−1	−1	2	30.13	33.51
2	−1	1	−1	1	45.44	42.50
3	1	−1	−1	1	76.12	75.67
4	−1	−1	1	1	48.84	43.14
5	−1	−2	1	−1	35.46	36.02
6	1	−1	1	1	51.60	54.63
7	1	1	−1	−1	45.33	51.13
8	1	1	1	1	84.77	81.51
9	0	0	0	0	76.51	76.53
10	0	0	0	0	78.48	76.53
11	0	0	0	0	77.53	76.53
12	0	0	0	0	75.69	76.53
13	0	0	0	0	74.91	76.53
14	0	0	0	0	76.12	76.53
15	−1	−1	−1	1	43.64	42.21
16	−1	1	−1	−1	31.00	27.62
17	1	−1	−1	−1	50.11	52.90
18	−1	−1	1	−1	37.43	38.33
19	1	1	−1	1	81.29	80.08
20	−1	−1	1	1	50.11	47.01
21	1	−1	1	1	70.44	73.51
22	1	1	1	−1	55.34	56.45
23	0	0	2	0	68.26	69.81
24	0	0	−2	0	64.96	63.56
25	2	0	0	0	80.02	74.43
26	−2	0	0	0	14.81	20.54
27	0	2	0	0	37.79	40.87
28	0	−2	0	0	41.71	38.77
29	0	0	0	−2	45.41	38.19
30	0	0	0	2	64.57	71.95

### Kinetics of accelerated storage study

The shelf-life prediction of foodstuff is based on environmental circumstance viz., temperature, humidity, microbes, etc., and its reaction kinetics. Under these environmental circumstances, the temperature is decisive to influence the storage kinetics. At optimum condition storage kinetics of SFE extract was conducted as are in the model suggested by Dien *et al*.^38^. The extract was stored in transparent and amber-colored 30 mL airtight vial at 45 °C and 55 °C in incubator until it degraded up to 80%. The frequency of analytical testing is the next important decision. The higher the storage temperature, the more frequent should be the testing.

## Results and Discussion

**Figure 2:**
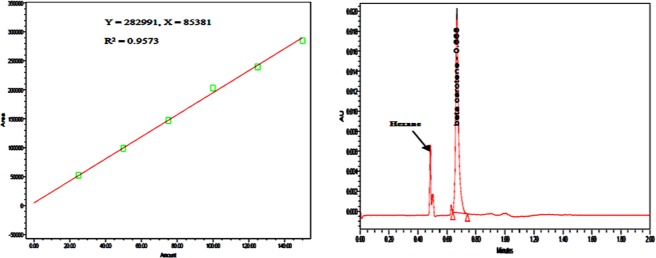
Supercritical fluid chromatography based UPC^2^ calibration curve (**A**) used in quantification of β-carotene extracted at the optimized condition and its chromatogram (**B**).

During SFE, the moisture content of lyophilized pericarp powder was 6.45% (d.b.). The quantitative analysis of β-carotene content was studied by supercritical CO_2_ based UPC^2^ system using the standard calibration curve of 0, 25, 50, 75, 100, 125 and 150 ppm (Fig. 2). Initially, β-carotene in ripe fresh pericarp was 7.63 mg/100 g, however, enzymatic digestion increased it up to 21.05% (9.72 mg/100 g) and 85.54 mg/100 g in digested dried powder. Enzymatic treatment improved the β-carotene content due to degradation of their interfacial tension, which increases its availability^11,34^. The reported results were in agreement with Lenucci *et al*.^34^ for tomato processing waste in which enzymatic treatment increased lycopene recovery up to ~150%. Vuong and King^39^ also reported the similar content of β-carotene in fresh *Momordica* genus ripe fruit (gac fruit) was 8.3 to 76.9 mg/100 g. However, Tran *et al*.^40^ reported 37.9.mg/100 g in gac powder.

### Extraction optimization

The data pertaining to the independent and response variables were analysed to get a regression equation with linear, square and interaction coefficients as follows:

2 $$\begin{array}{ccc}Y & = & -360.262743+0.837487{X}_{1}+4.973891{X}_{2}+3.890651{X}_{3}+0.621753{X}_{4}\\  &  & \,+\,0.001374{X}_{1}{X}_{2}-0.001029{X}_{1}{X}_{3}+0.001042{X}_{1}{X}_{4}+0.008958{X}_{2}{X}_{3}\\  &  & \,+\,0.003432{X}_{2}{X}_{4}-0.002163{X}_{3}{X}_{4}-0.001291{X}_{1}^{2}-0.09177{X}_{2}^{2}\\  &  & \,-\,0.024613{X}_{3}^{2}-0.002650{X}_{4}^{2}\end{array}$$

**Figure 3:**
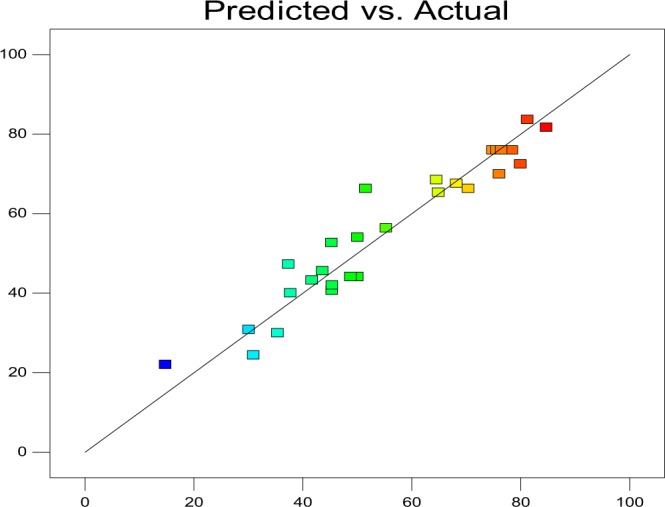
A plot of predicted and experimental value for the % yield of β-carotene extracted from ripe bitter melon pericarp using SFE technique.

**Figure 4:**
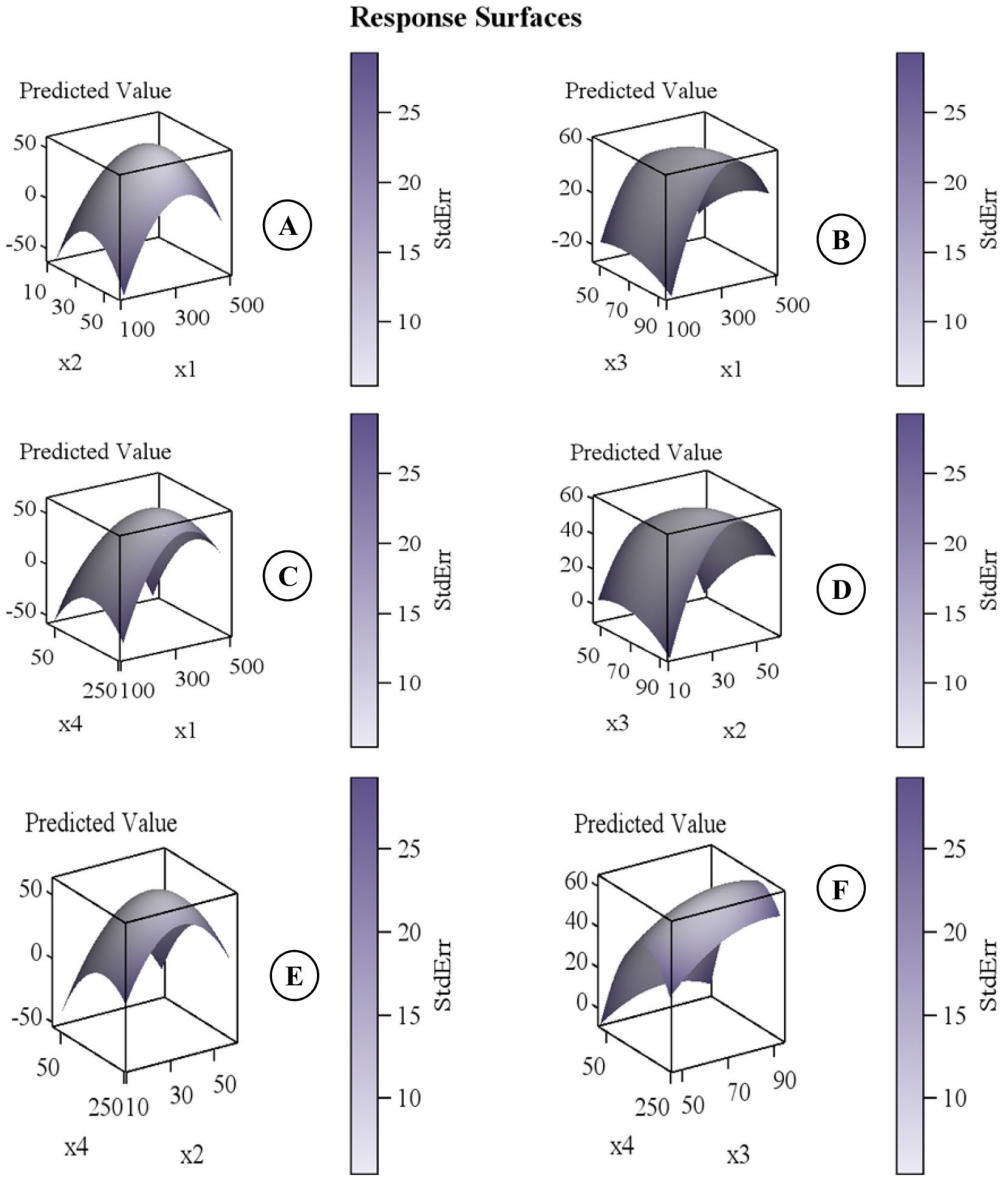
Response surface plot showing effects of independent variables on % yield (**A**–**F**) from ripe bitter melon pericarp while the remaining were kept at the central point (pressure: X_1_ - 300 bar; flow rate: X_2_ - 35 mL/min; temperature: X_3_ – 70 °C; and time: X_4_ - 135 min).

The predicted values of β-carotene content were calculated using the regression model and compared with experimental values. The value for the coefficient of determination (R^2^) was 0.966 which indicates the adequacy of the applied model. The statistical analysis of data revealed that linear, quadratic and model were significant (Table 3). The ANOVA also showed that there was a non-significant (p > 0.0019) lack of fit which further validates the model. The scattered plot between the experimental values and difference between the experimental and predicted values did not show a pattern that further indicated the adequacy of the model (Fig. 3). The levels of independent variables for optimal extraction conditions of β-carotene content were determined using response surface graphs plotted between each independent variable (Table 3). Variation in extraction pressure (X_1_) showed the most significant effect results in an increase in β-carotene recovery. Maximum β-carotene content was obtained at 393.32 bar; however, further increase in pressure up to 450 bar showed negative effect in % yield (Fig. 4A). Increase in pressure beyond a critical limit decreases the diffusion ability of supercritical CO_2_ mainly because of the enhanced compaction of the samples at higher pressure leading to chainaling of the supercritical CO_2_ around it rather than diffusing through it^41,42^. Kaur *et al*.^20^ reported similar trends for SFE of β-carotene from tray dried carrot. Results in case of flow rate (X_2_) was observed as list significant change in β-carotene yield; however, at a fixed flow rate of 35 mL/min was found to be the best. The increase in flow rate beyond 35 mL/min reduces yield significantly. This may be either the enhanced rate of dissolution of solute into the solvent or solvent might have passed touching the sample rather than penetrating inside it, because of the reduced solute-solvent interaction and dwell time of the sample in the extraction vessel as described by Topal *et al*.^43^. Temperature is an important consideration in any extraction on SFE. As an expected increase in temperature up to about 70 °C increases β-carotene yield at any given pressure since higher temperatures promote the solubility of solute and increase the % yield by the high mass transfer of solute in the matrix^44^. However, the same reduces drastically with any further increase in temperature beyond 70 °C. This can be explained by the loss of balance between the supercritical CO_2_ density and the solute vapor pressure. The effect of temperature at any given time period also remains the same up to a threshold value beyond which the extraction yields have significantly reduced (Fig. 4B). This could be due to the adverse effect of temperature leading to β-carotene degradation and isomerization as suggested by Gomez-Prieto *et al*.^45^ and Nobre *et al*.^46^. Extraction time, in fact, decides the amount of supercritical CO_2_ available for the extraction process. In case the available supercritical CO_2_ is a limiting factor, the completeness of extraction is adversely affected^47^. However, the increase in time period beyond a point where the available supercritical CO_2_ suffices the completeness of extraction could lead to detrimental effect because of other controlling parameters like temperature. Pressure level as described earlier could play a significant role either in aiding extraction by solvent densification or limit it because of sample compaction^45,47^. Hence, an appropriate balance between the two factors is essential for maximization of β-carotene yields (Fig. 4C). This is further strengthened by the surface plot between the temperature of extraction and the flow rate of supercritical CO_2_ (Fig. 4D). It can be clearly seen that increase in extraction time significantly enhances the β-carotene yields since it leads to enhance the time of the solvent with the solutes thereby enhancing the penetration and subsequent extraction of β-carotene from the sample matrix; however, increasing inflow rate of supercritical CO_2_ resulted in no significant effect (Fig. 4E). The interface between extraction time and temperature as shown in Fig. 4F was found to be significantly influenced by these independent variables. At initial extraction temperature increasing the time, resulting in an advantageous effect on β-carotene yield, however, a further increase in the temperature beyond 70 °C lead to extensive degradation of thermo-sensible β-carotene resulted in the loss of yield^41,47^. Based on a statistical analysis of data using PROCRSREG of SAS, it was found that a maximum extraction efficiency of 90.12% of β-carotene could be achieved using 69.15 °C temperature, 393.31 bar pressure, 36.98 mL/min flow rate for 190.36 min (Table 4).

**Table 3:** Analysis of variance of (ANOVA) independent variables for the extraction of β-carotene from the ripe bitter melon pericarp.

Regression	Yield			
	DF	Sum of Squares	F-Value	Pr > F
Linear	4	6132.400179	65.36	<0.0001
Quadratic	4	3613.201070	38.51	<0.0001
Cross product	6	290.457584	2.06	0.1195
Total Model	14	10036	30.56	<0.0001
Lack of Fit	10	343.518284	20.66	0.0019
Pure Error	5	8.315508	—	—
Total Error	15	351.833792	—	—

Note**:** R-Squares 0.9661, Degree of freedom (DF).

**Table 4:** Canonical analysis of response surface based on coded data for TBC and yield.

Factor	Critical Value		Coding Coefficients	
	Coded	Un-coded	Subtracted off	Divided by
X_1_	0.62	393.32	300.00	150.00
X_2_	0.099	36.98	35.00	20.00
X_3_	−0.04	69.15	70.00	20.00
X_4_	0.62	190.36	135.00	90.00

Predicted value at the stationary point for % yield = 90.11.

### Confirmatory studies

**Figure 5:**
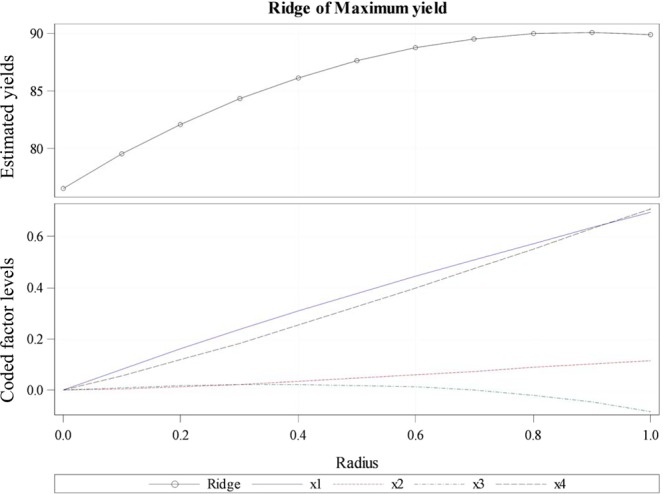
An estimated ridge of maximum response for variable % yield.

Additionally, three experimental runs were conducted at the optimum combination of independent variables to validate the same. The extraction yield obtained was 91.61%, 88.92% and 87.56% (mean value of 89.36 ± 0.68%) indicating good agreement with the results using statistical modeling. For justification of the above independent variables, the estimated ridge of maximum response for the dependent variable (% yield) shown in Fig. 5, revealed that the maximum yield was 90.099% at stationary point X1 = 395.002 bar, X2 = 37.03 mL/min, X3 = 69.03 °C and X4 = 191.66 min.

### Effect of storage temperature on storage stability

**Figure 6:**
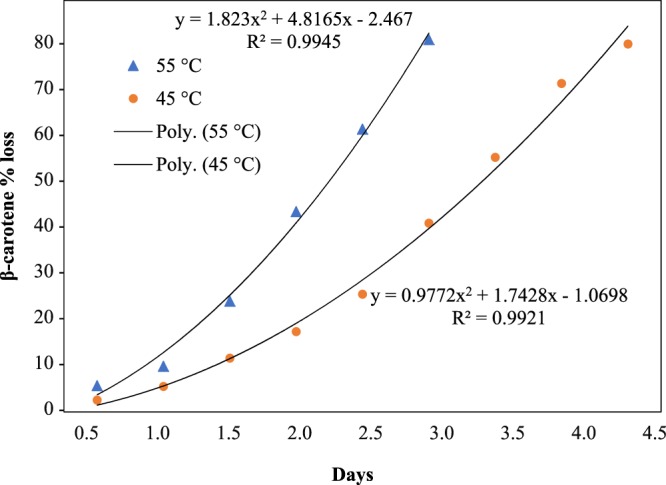
Effect of temperature 55 °C (**A**) and 45 °C (**B**) percentage loss and TBC extracted from ripe bitter melon pericarp at optimum condition.

In our experiments, we recognized quite clearly that total carotene decrease day by days when preserving β-carotene at 55 °C than 45 °C. Although all sample is kept in an incubator in airtight amber color vials, β-carotene owing to decomposition at high temperature, its bound energy goes from basic energy to excitation energy so molecule breakdown. At higher storage temperature the storage stability was 2.5 days with 90.48% loss than lower temperature (45 °C) 5 days with 89.41% loss (Fig. 6). Calculating from the above figure using polynomial equations, in order to get carotene 30%, it should keep within 3.09 days (55 °C) and 6.16 days (45 °C).

3 $${Q}_{10}=\frac{6.16}{3.09}=1.99$$

where Q_10_ is increase in the rate of the reaction when the temperature is increased by 10 °C during storage.

Storage duration at of β-carotene at 10 °C (Eq. (4)) and 5 °C (Eq. (5)) (carotene 30% reduction) will be:

4 $${F}_{2}={f}_{1}\times {Q}_{10}^{\frac{\Delta }{10}}=3.09{(199)}^{\frac{55-10}{10}}=68.36\,days\approx 2.27\,months$$

5 $${F}_{2}={f}_{1}\times {Q}_{10}^{\frac{\Delta }{10}}=3.09{(199)}^{\frac{55-5}{10}}=96.43\,days\approx 3.21\,months$$

where f_1_ - time between tests at the higher temperature, F_2_ - storage life at the lower temperature, Δ - difference in degrees centigrade between the two.

Therefore, we can keep β-carotene within 2.27 months at 10 °C or 3.21 months at 5 °C to maintain 70% TBC carotene. Retention of extracted β-carotene from gac fruit (*Momordica cochinchinensis* Spreng) stored at the same storage temperature was also agreeable with this study^38^.

## Conclusion

SFE of β-carotene from the ripe pericarp of *Momordica* genus has gained great attention in the current year. The study reviewed show that ripe bitter melon pericarp SFE-CO_2_ extracts are interesting, innovative, and high-quality products rich with β-carotene. Optimization of experimental parameters, such as pressure, CO_2_ flow rate, temperature and extraction period of enzymatically treated lyophilized ripe bitter melon pericarp matrix was done. The experimental values of β-carotene yield were varied from 14.81% to 84.77%. The statistical model revealed the thirty experiment to optimize the best extraction condition of SFE. The second-order model developed for β-carotene yield exhibited non-significant lack of fit and a high value for the coefficient of determination (0.9661). The surface graph indicated that maximum β-carotene % yield was obtained by extracting ripe bitter melon pericarp at 69.15 °C temperature, 393.31 bar pressure, 36.98 mL/min flow rate for 190.36 min. The expected storage stability of extracted β-carotene in the amber-colored vial to strictly restrict oxygen and light was 2.27 months at 10 °C or 3.21 months at 5 °C can maintain 70% of β-carotene.

### Ethical approval

**Informed consent:** This article does not contain any studies with either animals or human participants performed by any of the authors.
